# One-Port Coaxial Line Sample Holder Characterisation Method of Dielectric Spectra

**DOI:** 10.3390/s24175573

**Published:** 2024-08-28

**Authors:** Iman Farhat, Lourdes Farrugia, Julian Bonello, Rafel Grima, Raffaele Persico, Charles Sammut

**Affiliations:** 1 Department of Physics, University of Malta, MSD 2080 Msida, Malta; lourdes.farrugia@um.edu.mt (L.F.); charles.v.sammut@um.edu.mt (C.S.); 2 Department of Environmental Engineering DIAM, Università della Calabria, 87036 Rende, Italy; raffaele.persico@unical.it

**Keywords:** coaxial line, dielectric properties, material under test, sample holder, vector network analyser, dielectric measurements, microwave measurement

## Abstract

A technique for solving the one-port closed coaxial transmission line sample holder scattering equation for complex permittivity inversion for lossy materials is presented. A non-linear least-squares procedure is used for the determination of parameters for the specification of the spectral functional form of the complex permittivity. The method allows for accurate retrieval of many low- and high-permittivity dielectric materials in the frequency range of 1 GHz to 3 GHz inserted into the coaxial cell. Using this method, the complex permittivity of a number of liquids and a Maltese soil known as Bajjad soil have been extracted by measurements using a short terminated coaxial transmission line sample holder. The proposed novel inversion method is mainly based on the reflection coefficient of the test material. The measured results of the complex permittivity of liquid dielectrics such as ethanol, methanol, and TX100 are validated and compared with previously published data obtained from measurements made by the National Physical Laboratory (NPL) using a two-port measurement setup made with the same commercial coaxial transmission line sample holder used in the one-port setup. Since the technique allows broadband measurements, it has been used to characterise the soil dielectric spectrum in the frequency range of 1–3 GHz, which is also compared with results from a two-port setup of the same coaxial line. The experimental results are a validation of the proposed approach for different types of materials.

## 1. Introduction

The characterisation of dielectric properties of materials is a significant and continually evolving area of study, with extensive work published in recent decades in various fields [1,2,3], including but not limited to remote sensing and medical imaging. One of the most powerful techniques is the transmission line method, which is often used to characterise materials in the microwave region. This method provides accurate dielectric property measurements for a material under test (MUT) over a frequency range. Three common transmission line methods are used for wideband characterisation of material dielectric spectrum, known as coaxial open-ended probes [4], coaxial cells [5], and waveguide transmission-line cells [6].

The transmission line methods often rely on transmission/reflection and reflection techniques, which have undergone significant evolution and modifications to enhance accurate extraction of complex permittivity [7]. However, in the MHz and lower GHz frequency range, and in transmission line methods, waveguides tend to be very large in size and bulky [8]. Hence, coaxial cell techniques are preferable for the microwave frequency range. Open-ended coaxial probes, despite their capability to operate across a broad frequency spectrum, find limited application in soil measurements due to the inherent non-uniformity of soil. Nevertheless, coaxial transmission-line cells have emerged as a preferred choice for characterising soil dielectric spectra. The preference stems from their ability to accommodate a wider range of soil types, leading to more reliable and versatile soil dielectric measurements. This method involves enclosing the MUT within a coaxial cell for analysis. Every method for characterising the dielectric spectrum of materials using a coaxial transmission-line cell relies on a vector network analyser (VNA) to measure the scattering parameters of the samples. Hence, the present work proposes a one-port VNA approach to extract dielectric properties based on a single measurement of the $S_{11}$-parameter, relying only on the reflection method. The application of a one-port coaxial cell terminated in a short significantly lowers the cost of the system in comparison to two port setups.

The objective of this paper is to demonstrate the validity of a proposed novel approach using a coaxial sample holder terminated by a short for the determination of the dielectric properties of a MUT by a single one-port measurement of the scattering $S_{11}$ parameter in the microwave range. The procedure of retrieving the real and imaginary parts of the complex permittivity ($\varepsilon_{r}=\varepsilon_{r}^{\prime }-j\varepsilon_{r}^{\prime \prime }$) relies on minimising the difference between the measured and predicted $S_{11}$ parameters. This is achieved by identifying the parameters necessary to define the spectral functional form of the complex permittivity. The methodology is verified based on measurements of reference materials such as methanol, ethanol and TX100, for their permittivity values can be obtained from the table presented by the National Physical Laboratory (NPL) [9], using a commercial coaxial sample holder transmission line available at the Electromagnetics Research Group (EMRG) laboratory within the Department of Physics at the University of Malta. The findings of the proposed one-port method are also validated by employing the two-port reflection transmission method discussed in [10,11,12] on a coaxial line sample holder. Furthermore, the one-port permittivity inversion method is used to characterise the dielectric properties of a soil sample available at the CST Microwave Studio material library, a PTFE material machined to fit in the coaxail line, and a soil sample obtained from the Maltese Islands, known as Bajjad soil [6], which, in turn, is validated using a two-port reflection transmission method setup of the same commercial coaxial cell. The measurement of dielectric properties of soil is a helpful technique to estimate soil moisture content in addition to its potential to determine the pollution content in soil.

This paper first presents the derivation of the mathematical model of the closed-coaxial cell scattering equation in Section 2. Then, Section 3 details the numerical method for solving the transmission line model in an iterative approach and discusses the measurement setup. The measurement results of standard liquids and soil are presented in Section 4. Then, the discussion of the results and uncertainty analysis is performed in order to assess and improve the accuracy of the one-port coaxial cell terminated by a short in Section 5. Finally, in Section 6, we recapitulate all important points and main findings regarding the proposed method.

## 2. Theoretical Analysis

This section, a theoretical model has been developed to analyse the incident and reflected wave propagation inside the coaxial line sample holder, and retrieve the dielectric properties of a lossy material filling it. The model includes the geometry and electrical properties of a coaxial transmission line terminated at one of the ends with a short, and filled with the MUT between the short and the measurement plane.

### TEM Wave Propagation in the Presence of a Sample

**Figure 1:**
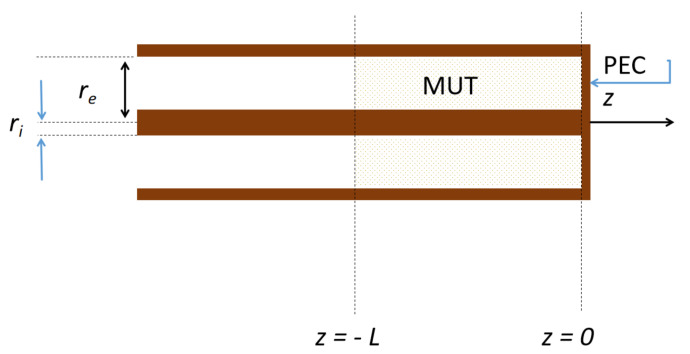
Schematic model of the coaxial transmission line cell.

The model of the coaxial transmission line cell is shown in Figure 1. The MUT fills the last part of the coaxial line sample holder, which is terminated with a perfect electric conductor (PEC) termination and is fed from the opposite end. The MUT extends from the abscissa z=−L up to the abscissa z=0, located at the short circuit. The response of a medium inside a coaxial transmission line can be found by transitioning from electric and magnetic fields to voltage, current, and impedance. The characteristic impedance of a coaxial line when void at z=−L is given in Equation (1) [13,14]:

(1) $$Z_{{l_{2}}_{0}}=\frac{1}{2\pi }\sqrt{\frac{\mu_{0}}{\varepsilon_{0}}}\ln\left(\frac{r_{e}}{r_{i}}\right),$$

where $r_{i}$ and $r_{e}$ are the radius of the centre conductor and the inner radius of the external conductor of the coaxial line, respectively, $\varepsilon_{0}=8.854\times 10^{-12}\;Fm^{-1}$ is the absolute vacuum dielectric permittivity and $\mu_{0}=4\pi \times \;10^{-7}\;Hm^{-1}$ the absolute vacuum permeability.

**Figure 2:**
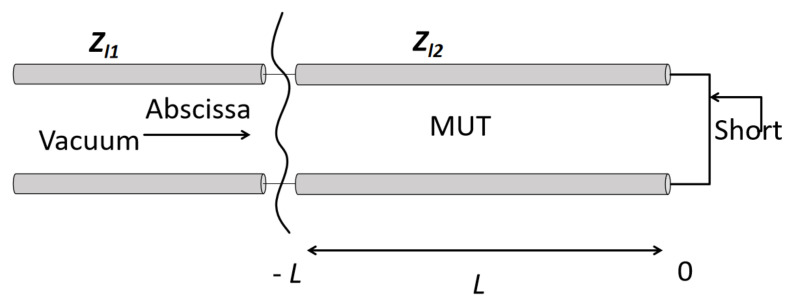
Schematic diagram of the coaxial TEM transmission line terminated in short.

In a coaxial transmission line, the flow of current and the difference in potential between the inner and outer conductors create electric and magnetic fields perpendicular to each other and transverse to the direction of propagation along the axis. This enables a Transverse Electromagnetic (TEM) wave to travel along the coaxial transmission line. The TEM wave propagation in the presence of a sample can be inferred by studying the schematic representation of the coaxial sample holder transmission line shown in Figure 2.

At the abscissa interface, immediately before the part filed with the MUT in Figure 2, a discontinuity occurs where the characteristic impedance changes from the source intrinsic impedance $Z_{l1}$ of the empty coaxial cable to the intrinsic impedance of the cable filled up with the MUT, $Z_{l2}$. The section of length *L* in Figure 1 represents the coaxial line part filled with the MUT and terminated in short at L=0. The characteristic impedance in this part depends on the factor $\sqrt{\frac{\varepsilon_{0}\varepsilon_{R}}{\mu_{0}\mu_{R}}}$, yielding:

(2) $$Z_{l_{2}}=\sqrt{\frac{\mu_{R}}{\varepsilon_{R}}}Z_{{l_{2}}_{0}},$$

where $\mu_{R}$ and $\varepsilon_{R}$ are the intrinsic, possibly complex , relative permeability and permittivity, respectively, of the MUT. Hence, for a lossy medium, the impedance of the load transformed to −L can be determined as [15]:

(3) $$Z\left(-L\right)=Z_{l_{2}}\left[\frac{Z_{L}+Z_{l_{2}}\tanh\gamma L}{Z_{l_{2}}+Z_{L}\tanh\gamma L}\right].$$

For a lossy line terminated by a short, $Z_{L}=0$ and $\gamma =\alpha +j\beta =jk_{c}$, where $k_{c}$ is the wavenumber, and can be represented as $k_{c}=k_{0}\sqrt{\mu_{R}\varepsilon_{R}}$, where $k_{0}$ is the wavenumber in free space $\left(k_{0}=\frac{\omega }{c_{0}}\right)$, $c_{0}={\left(\sqrt{\varepsilon_{0}\mu_{0}}\right)}^{-1}=3\;\times \;10^{8}$ m/s is the speed of light in free space, and ω the angular frequency. Hence, $\tanh\left(jk_{c}L\right)\;=j\tan\left(k_{c}L\right)$. So, Equation (3) reduces to:

(4) $$Z\left(-L\right)=-j\sqrt{\frac{\mu_{R}}{\varepsilon_{R}}}Z_{{l_{2}}_{0}}\tan\left(\frac{\omega }{c}\sqrt{\mu_{R}\varepsilon_{R}}L\right).$$

The reflection coefficient $S_{11}\left(-L\right)$ immediately before the MUT can be determined as:

(5) $$\begin{array}{cc} S_{11}\left(-L\right) & =\frac{jZ_{l_{20}}\sqrt{\frac{\mu_{R}}{\varepsilon_{R}}}\tan\left(k_{0}\sqrt{\mu_{R}\varepsilon_{R}}L\right)-Z_{l_{1}}}{jZ_{l_{20}}\sqrt{\frac{\mu_{R}}{\varepsilon_{R}}}\tan\left(k_{0}\sqrt{\mu_{R}\varepsilon_{R}}L\right)+Z_{l_{1}}}. \end{array}$$

(6) $$\begin{array}{cc} \; & =\frac{jZ_{l_{20}}\sqrt{\frac{\mu_{R}}{\varepsilon_{R}}}\sin\left(k_{0}\sqrt{\mu_{R}\varepsilon_{R}}L\right)-Z_{l_{1}}\cos\left(k_{0}\sqrt{\mu_{R}\varepsilon_{R}}L\right)}{jZ_{l_{20}}\sqrt{\frac{\mu_{R}}{\varepsilon_{R}}}\sin\left(k_{0}\sqrt{\mu_{R}\varepsilon_{R}}L\right)+Z_{l_{1}}\cos\left(k_{0}\sqrt{\mu_{R}\varepsilon_{R}}L\right)}. \end{array}$$

Let us now focus on non-magnetic materials ($\mu_{R}=1$), which is the case in most geophysical surveys. Using Euler’s formulas and some algebraic manipulation, the reflection coefficient at z=−L can be simplified as [13,16,17]

(7) $$S_{11}\left(-L\right)=\frac{\left(\frac{Z_{l_{20}}}{\sqrt{\varepsilon_{R}}}-Z_{l_{1}}\right)-\left(\frac{Z_{l_{20}}}{\sqrt{\varepsilon_{R}}}+Z_{l_{1}}\right)e^{jk_{0}\sqrt{\varepsilon_{R}}L}}{\left(\frac{Z_{l_{20}}}{\sqrt{\varepsilon_{R}}}+Z_{l_{1}}\right)-\left(\frac{Z_{l_{20}}}{\sqrt{\varepsilon_{R}}}-Z_{l_{1}}\right)e^{jk_{0}\sqrt{\varepsilon_{R}}L}}.$$

The proposed technique for the solution of the one-port scattering equation for complex permittivity determination is based on the nonlinear regression procedure. Once the mathematical model of the reflection coefficient ($S_{11}\left(-L\right)$) is expressed as a function of $\varepsilon_{R}$, it can be inverted to yield the complex relative permittivity from the measured reflection coefficient, as explained in the following section.

## 3. Numerical Algorithm

The procedure for the extraction of material parameters involves fitting algorithms to minimise the distance between the calculated reflection coefficient from the derived mathematical model (Equation (7)) and the corresponding measured quantities. This can be conducted over the whole frequency range or on a point-by-point basis (i.e., at individual frequency points). Given the computational impracticality of treating all permittivities as unknowns across observation frequencies, the proposed numerical model uses not only an explicit frequency-dependent formulation [11], but also satisfies causality requirements. Hence, this study employs the Debye model, a truncated Laurent series expansion, to retrieve permittivity values for nonmagnetic materials. The permittivity is expressed as a function of frequencies, as follows [11]:

(8) $$\varepsilon_{R}^{*}\left(\omega \right)=A_{0}+\sum_{i=1}^{4}\frac{A_{i}}{1+j|B_{i}|\omega }+\sum_{i=5}^{8}\frac{A_{i}}{\left(1+j\right|B_{i}{\left|\omega \right)}^{2}},$$

where $A_{i}$ and $B_{i}$ are real numbers. The algorithm finds the parameters $A_{0}$, $A_{i}$ and $B_{i}$ that provide the best fit of the reflection coefficient of the model function to the measured $S_{11}$ scattering parameter. This is achieved by minimising the sum of squares of the differences between the model function and measured $S_{11}$-parameters;

(9) $$min\|\sum (S_{11}\left(\omega \right)-P_{11}\left(\omega \right))\|$$

where $S_{11}\left(\omega \right)$ and $P_{11}\left(\omega \right)$ (Γ(−L)) are the measured and predicted $S_{11}$-parameters vectors, respectively. The minimisation is based on the Least Square fit, which is a procedure that finds the best fit of a non-linear curve by minimising the sum of the squares of the offsets of the points in the curve.

However, the success of the extraction of the dielectric properties also depends on the proper choice of dispersive laws for the MUT. For low permittivity materials, the fitting algorithms may experience nonconvergence issues, or the parameters of the models may be determined with excessive errors using the expanded truncated Laurent series expansion. The proposed method uses the Cole-Davidson model for low permittivity materials and to cross-validate the results from the Laurent series expansion. This is an empirical modification of the single-pole Debye relaxation model;

(10) $$\varepsilon_{R}^{*}\left(\omega \right)=\varepsilon_{\infty }+\frac{\varepsilon_{s}-\varepsilon_{\infty }}{{\left[1+\left(j\omega \tau \right)\right]}^{\beta }},$$

where $\varepsilon_{s}$ and $\varepsilon_{\infty }$ are the values of the real part of the complex relative permittivity at low and high frequency, respectively, τ is the relaxation time, and β is a positive real constant (0≤β≤1).

Two MATLAB https://www.mathworks.com (accessed on 1 April 2023) codes were developed using the nonlinear least square curve fitting approach. Both were aimed at searching for minimum values, considering constraints that avoid local minima. The first code used a deterministic numerical technique, known as the Levenberg–Marquardt (LM) algorithm, developed in-house according to [18]. The LM algorithm was chosen because it is a popular optimization technique for solving nonlinear least squares problems. It incorporates aspects of the Gauss–Newton algorithm and gradient descent. Additionally, it is more robust than the Gauss–Newton method alone, especially when the initial guess is far from the optimal solution. The in-house LM code was developed to retrieve the permittivity of liquid samples, adhering to the Laurent series dispersion model. The developed algorithm automatically scales the parameters by default, given initial estimates of the variables $A_{i}$ and $B_{i}$ in Equation (8).

The second code employs MATLAB’s built-in nonlinear least squares curve fitting routine using lsqnonlin syntax. This procedure was developed for the Cole-Davidson model dispersion mode, which is implemented to rely on two built-in optimisation conversions from two algorithmic options: the ’interior-point’ algorithm or the ’Levenberg–Marquardt’ method, where the solution from both options converges to the same solution.

In both codes, the approach iterates until the difference between observed and predicted values is sufficiently small, or a specified number of iterations is reached within the desired accuracy. The inversion of the relative complex permittivity is sensitive to the trial initial values.

## 4. One-Port Measurement Setup

**Figure 3:**
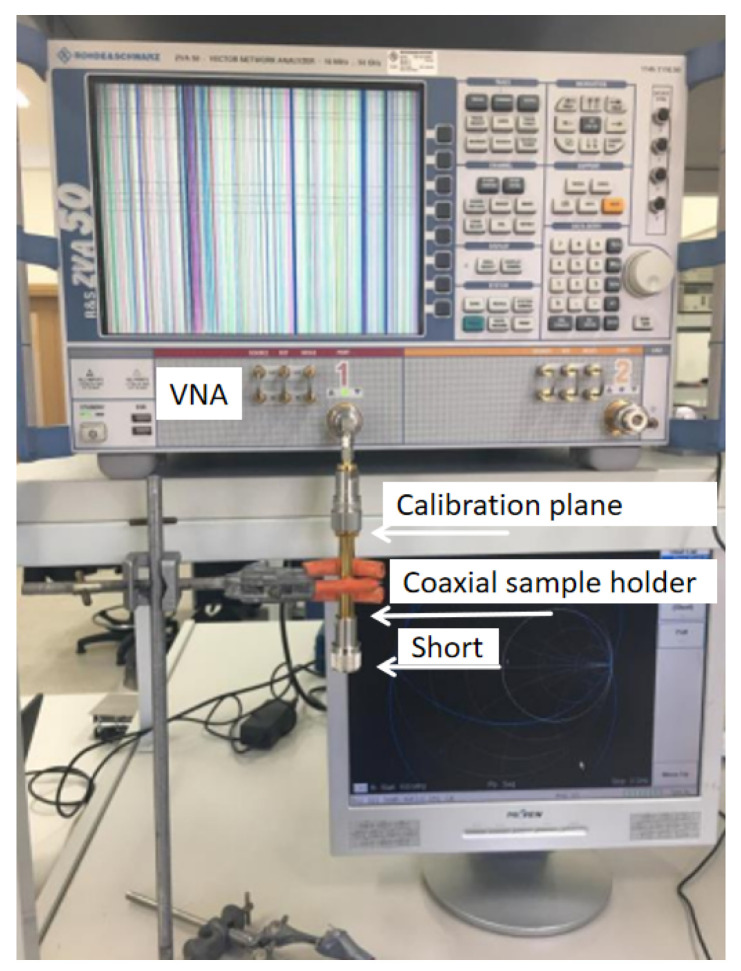
Experimental setup showing the closed coaxial transmission line attached to the VNA.

The experimental setup consists of a two-port Rohde and Schwarz ZVA-50 10 MHz– 50 GHz Vector Network Analyzer (VNA) connected to a closed-coaxial transmission line, as depicted in Figure 3. The frequency range for the measurements of the $S_{11}$-parameters of the material under test (MUT) was from 1 GHz to 3 GHz.

**Figure 4:**
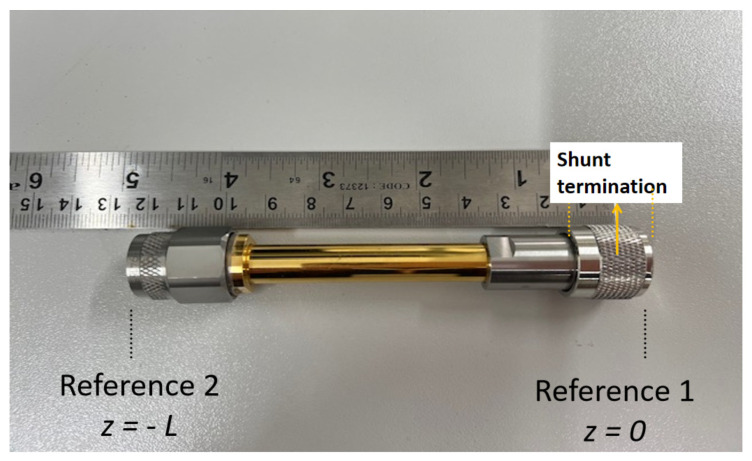
The closed coaxial transmission line and the point z=−L at which the calibration was conducted.

The closed coaxial transmission line is shown in Figure 4. It is essentially a 5.4 cm coaxial line section with N-type connectors at the two ends, having a total length of around 11.2 cm. The coaxial line outer diameter is 3.25 mm and its inner radius is 1.55 mm. The material under test (MUT) was placed inside the coaxial transmission line sample holder, and tightly sealed using thin tape, to ensure no liquid leakage risk to the VNA port. The VNA was connected to the coaxial transmission line via a coaxial cable connected to Port 1 at reference plane 2 (z=−L), as indicated in Figure 3, being shorted at the end referred to as reference plane 1. The top part (reference 2) of the closed coaxial transmission line was connected to Port 1 of the VNA using an adapter and a SM3016 connector, at which the line was calibrated. The setup was calibrated using a standard calibration kit (open, short, load) at the calibration plane shown in Figure 3. The calibration was always verified by examination of the Smith chart displays for the ‘open’, ‘closed’ and ‘load’. The other end of the closed coaxial transmission line was connected to a N-type short.

### 4.1. Two-Port Setup

**Figure 5:**
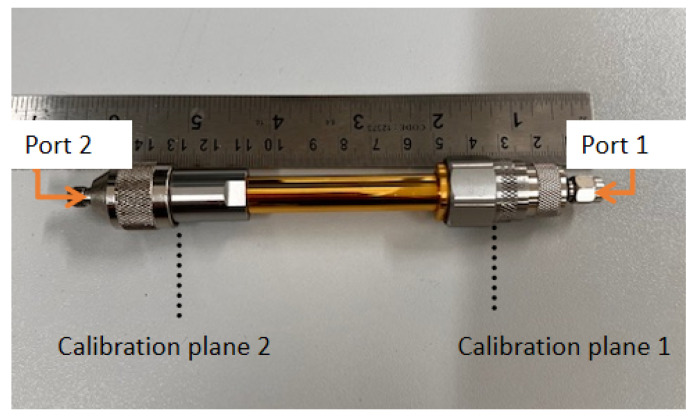
The commercial two-port coaxial transmission line.

In the two-port measurement setup scenario, a commercial coaxial line is connected to two connectors, shown in Figure 5, which are then linked to the VNA. The VNA is calibrated using through, open, short, and termination standards at each port. The measured scattering (S) parameters are subsequently recorded and saved for analysis.

The procedure for the extraction of the material parameters involves minimising the distance between the calculated scattering parameters, with their corresponding measured values using a fitting algorithm. The fitting algorithm utilises the built-in MATLAB nonlinear least square curve fitting using the lsqnonlin syntax. The minimisation is carried out over the whole frequency range using the two-port traditional scattering formulation of the standard Baker-Jarvis iterative method presented in [11,19] for a coaxial sample holder transmission line. The objective function to be minimised for the nonlinear least-squares-type fitting is given in [20].

### 4.2. Materials Under Test (MUT)

Initially, the aim of the project was to standardise the coaxial transmission line dielectric measurement method using standard liquids. Ethanol, methanol, and TX100 were selected for this purpose, with the aim of ultimately characterising the dielectric spectrum of a soil using the closed coaxial transmission line method.

To validate measurements obtained through the proposed one-port coaxial transmission line method, results were compared to the standard complex permittivity of the liquids provided by NPL [9]. A crucial aspect of the proposed method involves utilising a coaxial cell terminated in short, enabling the extraction of sample $S_{11}$ scattering parameters. Subsequently, these $S_{11}$ parameters are inputted into a dielectric permittivity extraction algorithm. This approach ensures rapid and consistent measurements whilst eliminating the need for flexible microwave cables.

#### 4.2.1. Standard Liquids

A micropipette was used to pour the liquid of choice into the coaxial transmission placeholder, filling it up to the very top. Preparing the closed coaxial transmission line for such tests required a more careful procedure. It was essential in the tests to keep the volume of the liquid inside the closed coaxial transmission line constant without any evident leakages. This would have caused changes in the volume of the liquid and possible inconsistencies in the $S_{11}$ measurements. The thinner end of the closed coax was taped using very thin layers of electrical tape, requiring at least five turns. When the cap was screwed back on, the outer layer was taped too, thus making sure that no liquid seeped through. The taping was tested out by pouring the liquid into the hollow cylinder of the closed coax. After making sure that this end was sealed, the central conductor was placed at the centre of the closed coax and the test liquid poured using a pipette. For each liquid, a new pipette was used to avoid cross contamination. The cap was put on at the other end of the closed coax, taping it on the outer side only. Measurements were taken by placing the short at the respective end. Finally, the closed coaxial transmission line was connected to the VNA as shown in Figure 3, and the measurements were recorded. This was repeated a further two times for a total of three repeated measurements. The liquid and the end of the pipette was replaced for each measurement.

#### 4.2.2. Soil

The second phase of measurements examined soil as the MUT, which included Bajjad soil, a type of soil which is found on the Maltese Islands. The Bajjad soil consists of 26% clay, 39% silt, and 35% sand. The soil dielectric measurements were taken for two different moisture levels. The normal Bajjad soil and hydrated Bajjad soil obtained by hydrating 9 g of the soil with 3 mL water. Two $S_{11}$ measurements were recorded for each sample.

## 5. Results

In this section, the measured and calculated permittivity results for different liquids and soil (of nonmagnetic characteristics, $\mu_{R}=1$) are presented. Comparison of the proposed method solution to data obtained from NPL sheet [9] for standard liquids is displayed. Each liquid had three repeated measurements. The $S_{11}$ parameters were extracted from the VNA and plotted to visually identify if the trends were consistent. A MATLAB program was developed, which aimed to (a) average the measurement data, (b) calculate the standard deviation, and (c) plot the results. The permittivity inversion of the MUTs using the $S_{11}$ parameters measured from the VNA is conducted through a MATLAB in-house-code, developed based on the numerical algorithm discussed in Section 3.

This section is subdivided into two subsections. The first illustrates the results for the extraction of the standard liquids dielectric properties, and the second is related to the Bajjad soil permittivity retrieval for samples with different moisture content.

### 5.1. Liquids

**Figure 6:**
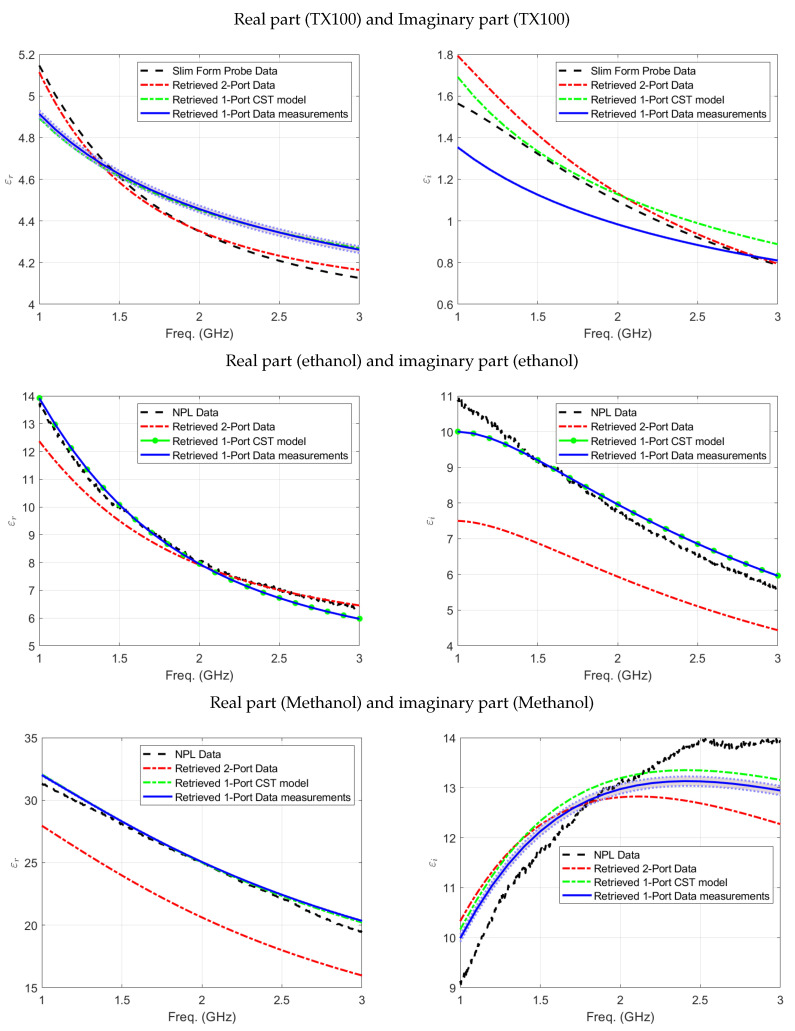
Real (**left** panel) and imaginary (**right** panel) parts of the retrieved permittivity as a function of the frequency of TX100, ethanol, and methanol, respectively.

$S_{11}$ parameter measurements of the three different liquids—ethanol, methanol, and Triton X-100 (TX100)—were made using the closed coaxial transmission line material holder. The aim of these measurements was to verify that the complex permittivity retrieved through the one-port proposed method were comparable to those derived from NPL data [9], two-port measurements of the same coaxial line, and measurements using the slim form open-ended coaxial probe. Three repeated measurements were taken for each liquid, and the mean and standard deviation were calculated. The standard deviation of the retrieved permittivity was plotted with the average of the extracted permittivity for the frequency range, ensuring repeatability in the measurements. Figure 6 shows the complex permittivity of the three standard liquids (TX100, ethanol and methanol, respectively), measured using the one-port coaxial sample holder measurement setup, compared to its CST Microwave Studio simulation model results, in addition to the results obtained through the two-port coaxial line measurement setup and those obtained using the slim form probe or extracted from the NPL data sheet values.

These figures highlight the consistency of the three measurement results conducted using the one-port approach for each liquid, as illustrated in the shaded areas of the graphs. Furthermore, the real part of the complex permittivity for all presented liquids shows good agreement with the values extracted from the NPL data sheet, the slim form open-ended coaxial probe measurement, and the two-port measurement setup. However, the imaginary part of the complex permittivity displays good agreement for methanol across all depicted inversion methods when compared to the NPL data values. On the other hand, for ethanol and TX100, the patterns are comparable, with ethanol showing much better agreement. These findings underscore the reliability of the one-port approach for measuring the complex permittivity across different liquids. The difference observed in the imaginary part between the one-port measurement and simulation results is attributed to potential uncertainties in filling the coaxial line to the end, which corresponds to variations in the sample length, particularly in the imaginary part, as it involves energy dissipation that is highly dependent on the exact physical configuration of the sample within the line.

### 5.2. Soil

**Figure 7:**
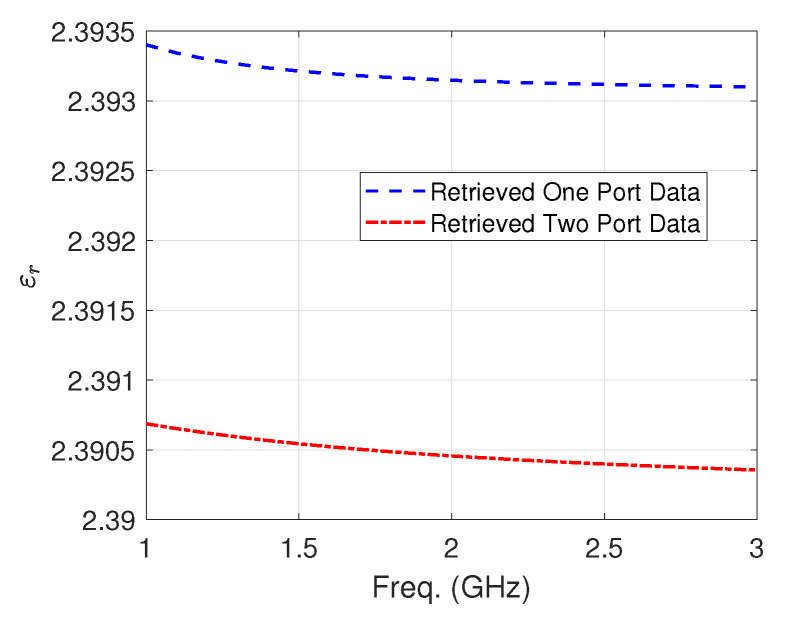
Comparison of the complex permittivity retrieved using the proposed method and PTFE data extracted from two-port coaxial cell data measurement as reference.

**Figure 8:**
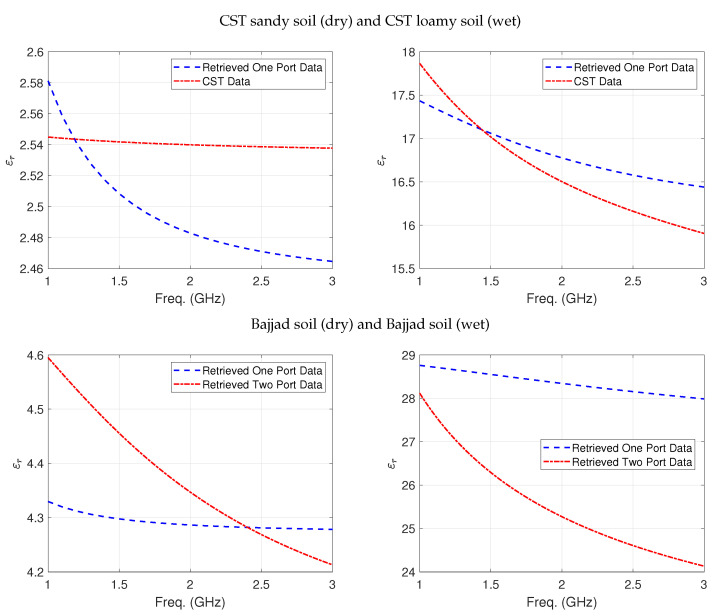
Retrieved permittivity of dry (**left** panel) and hydrated (**right** panel) CST Microwave Studio material library soil and Bajjad sample, respectively, as a function of frequency.

In the soil measurements, retrieval of the permittivity was first based on the initial guess from the inversion of one-port-based PTFE measurements (Figure 7) and soil data from the CST Microwave Studio material library and simulation (Figure 8).

Complex dielectric permittivity spectra of Bajjad soil samples measured with the one-port system and the spectra of the Bajjad hydrated soil sample content are depicted in Figure 8, respectively. It is as expected: moisture content heavily influenced the dielectric permittivity at all frequencies.

## 6. Discussion

A one-port optimisation method, based on shunt termination of a coaxial cell transmission line, has been presented as the solution to complex permittivity inversion, and appears to be a viable alternative to the two-port S-parameters technique. The technique allows a stable solution for a broad range of frequencies. Unlike the two-port method, which requires measurements of two S-parameters, the optimised one-port solution can obtain complex permittivity from one-port S-parameters at a single position, although the solution may exhibit some alternative minima.

The source of error in the one-port coaxial sample holder measurements is attributed to errors in the measurement of the $S_{11}$ parameter, the gap between the sample and the top of the sample holder connector to the VNA, and uncertainty in the sample length and connector mismatch. The error due to the gap around the sample and the sample length is accounted for by taking three measurements of the sample and inserting the MUT into the coaxial sample holder three times. The $S_{11}$ parameter is measured each time and the measurements are then averaged to give the $S_{11}$ data of the MUT. However, as discussed by Hill [11,21], these uncertainties are generally much smaller than the systematic uncertainty introduced by the network analyser.

$S_{11}$-parameters of low permittivity materials, such as TX100, PTFE, and soil, exhibit a periodic pattern. This indicates that there is more than one solution to the problem. Each root has a neighbourhood around it, within which convergence will occur based on the initial estimates made in that region. Hence, the existence of alternative optima in the mathematical model means that a reasonable initial guess is required to converge to the correct solution. The robustness of the proposed algorithm is based on adding constraints to the optimisation to enhance the convergence and accuracy of the solution. The constraints that were employed address the solution within upper and lower boundaries to the initial guess of Debye equation parameters. In addition to searching for minimum residuals converged from different iterations of different initial guesses, the robustness of a mathematical procedure is determined by how effectively the algorithm handles the region around the true root. As shown in Table 1, the one-port method generally produces comparable RMSE values compared to the two-port method across different materials.

## 7. Conclusions

The paper outlines a procedure for extracting the electric parameters of dispersive materials by inserting the material under test (MUT) inside a terminated coaxial transmission line sample holder, and exciting it with a vector network analyser from the other end to measure the reflection coefficient. The coaxial line section is terminated with a short circuit at one end. The inversion of the complex permittivity of the MUT is based on an iterative approach that implements a least-square minimization of the cost function, involving the measured and modelled reflection coefficients, with a regression algorithm over the entire frequency measurement range. The dielectric properties of standard liquids obtained using the one-port coaxial sample holder show good agreement with published NPL results. The inversion algorithm was also validated using data extracted from CST Microwave Studio simulation results of the coaxial cell CST Microwave Studio replicate model. Furthermore, the results achieved by the one-port coaxial transmission line model terminated in short were compared with the two-port coaxial sample holder technique’s results. The comparison indicates that the electric properties of a material can be determined precisely with this technique, with very good results in some cases and less good in others, especially regarding the imaginary part. This variability can be attributed to uncertainties in sample length and alignment within the cell for both coaxial-cell-based systems, significantly affecting measurement accuracy. Additionally, potential calibration errors may contribute to discrepancies. The experimental results of soil permittivity measurements are also presented to validate the proposed method on soil, as the dielectric properties are crucial for understanding the water content behaviour within the soil. This method can be used for assessing and evaluating soil pollution in the field. Overall, the method allows for the determination of various low- and high-permittivity dielectric materials, whether in the low- or high-loss range.

## Figures and Tables

**Table 1:** RMSE comparison of the different materials using the one-port and two-port inversion methods.

Material	One-Port RMSE	Two-Port RMSE
TX100	0.3982	0.4363
Ethanol	0.3378	0.3344
Methanol	0.4472	0.3896
PTFE	0.189	0.105
CST sand soil (dry)	0.2831	N/A
CST Loamy soil (wet)	0.2831	N/A
Bajjad soil (dry)	0.5	0.4258
Bajjad hydrated	0.3	0.6493

## Data Availability

The authors will provide the raw data upon request.
